# Pasteur’s Response to Koch

**Published:** 1883-05

**Authors:** 


### Pasteur's Response to Koch.

Many of our readers will remember Koch's reply to Pasteur in No. 6 of the *Lancet and Clinic.* To-day we take from the *Deut. Medical Zeitung* the following extract which is a response by Pasteur. Koch's objection at the International Congress at Geneva was that Pasteur had not adduced anything new or scientific. Pasteur refers to the simple method of weakening virus by means of the action of oxygen which is found in the air, and next that he announced the discovery of new kinds of microbes, and the conditions necessary to weaken their virulency. Unjustly Koch makes the accusation that Pasteur spoke of a new kind of hydrophobia that had been produced by inoculating a rabbitt with saliva from a child who had died with hydrophobia.

Pasteur only said that he had produced a new disease by inoculating with the saliva from a person who had died with hydrophobia; and that the microbe found in the saliva had not been described by any one of his co-workers, Chamberland, Roux and Thuillier; he explained its action and relation to the disease above mentioned.

Koch makes a statement in which he claims that Vulpian and Sternberg were the first to describe the virulency of the microbes found in the saliva of healthy persons; that the disease produced by the saliva microbe and the septicemia produced by Davaine in rabbits are not identical.

Pasteur answers these charges by saying that the interpretations and views of Koch are lacking experimental support and are nothing more than mere suppositions. "Not to you, sir, is due the honor of having discovered the manner in which bacilli and vibriones generate, neither have you shown the peculiar manner of their formation. You do not recognize the way to maintain them in a dust like condition nor the length of time that they could exist."

Pasteur says that the etiological doctrine as initiated and taught by him (that contagious diseases are due to a microscopic organism which acts as virus) has been recognized and accepted by Davaine, Chameau, Obermaier, Klebs, Coze, Filtz, Traube and Lister, and that Koch cannot but follow these great names, and acknowledge his indebtedness to French scientists. Koch contradicts himself, or he has changed his opinion since 1881, when he pointed out the weakening of milzbrand virus as very fallacious, while at the present time he writes that one of the greatest achievements of science is our power to weaken milzbrand virus. With regard to the figures on the effect of milzbrand vaccinations, Koch has erred very much, as can readily be be proven by statistics from the department cure et Loire.—*Lancet and Clinic.* C. W. T.

